# *Paracoccidioides brasiliensis* Induces α3 Integrin Lysosomal Degradation in Lung Epithelial Cells

**DOI:** 10.3390/jof9090912

**Published:** 2023-09-08

**Authors:** Bruna Rocha Almeida, Bianca Carla Silva Campitelli Barros, Debora Tereza Lucas Barros, Cristina Mary Orikaza, Erika Suzuki

**Affiliations:** Department of Microbiology, Immunology, and Parasitology, Escola Paulista de Medicina, Universidade Federal de São Paulo, Ed. Antonio C. M. Paiva, São Paulo 04023-062, SP, Brazil; b.almeida10@unifesp.br (B.R.A.);

**Keywords:** α3 integrin, vesicular traffic, lysosomal degradation, epithelial cell, *Paracoccidioides brasiliensis*

## Abstract

Studies on the pathogen–host interaction are crucial for the understanding of the mechanisms involved in the establishment, maintenance, and spread of infection. In recent years, our research group has observed that the *P. brasiliensis* species interact with integrin family receptors and increase the expression of α3 integrin in lung epithelial cells within 5 h of infection. Interestingly, α3 integrin levels were reduced by approximately 99% after 24 h of infection with *P. brasiliensis* compared to non-infected cells. In this work, we show that, during infection with this fungus, α3 integrin is increased in the late endosomes of A549 lung epithelial cells. We also observed that the inhibitor of the lysosomal activity bafilomycin A1 was able to inhibit the decrease in α3 integrin levels. In addition, the silencing of the charged multivesicular body protein 3 (CHMP3) inhibited the reduction in α3 integrin levels induced by *P. brasiliensis* in A549 cells. Thus, together, these results indicate that this fungus induces the degradation of α3 integrin in A549 lung epithelial cells by hijacking the host cell endolysosomal pathway.

## 1. Introduction

*Paracoccidioides brasiliensis* is a thermodimorphic fungus and one of the etiological agents of a human systemic mycosis named paracoccidioidomycosis (PCM). This disease is widespread in Latin America, particularly in Brazil, which accounts for 80% of the confirmed cases [1]. Although, historically, PCM patients were predominantly rural workers [2], recently in Brazil, PCM outbreaks have garnered attention in areas that suffered deforestation and massive soil disturbance, as occurred recently in the State of Rio de Janeiro during a highway construction [3].

Over the last decades, several studies have demonstrated the importance of epithelial cell responses to an infection. Besides forming a physical barrier in the body, respiratory epithelial cells have various receptors that constantly monitor the inhaled air to detect pathogens. As a result, epithelial cells may release chemical mediators, such as cytokines and chemokines, that promote the recruitment and activation of immune cells at the site of infection [4]. Pathogens, in turn, may interact with the host cell receptors and consequently manipulate cell signaling pathways to subvert host defenses, survive, and establish an infection in the host [4,5,6].

Integrin family receptors are present in a diverse range of cells in the host. These receptors are heterodimers composed of two subunits (α and β) that are non-covalently associated and, in mammals, 18 α-subunits (1 to 11, IIb, D, E, L, M, V and X) and 8 β-subunits (β1 to β8) can combine to form one of the 24 different heterodimers described in the literature [7,8]. Integrins are involved in several cellular functions, such as cytoskeletal organization, cell proliferation, adhesion, differentiation, and migration and, during an infection, several integrins are involved in host cell responses to various pathogens [9,10,11]. Shiota et al. [12], for example, described that α3 integrin is essential for the establishment of the infection of liver cancer cells by the non-enveloped hepatitis E virus (HEV). α5β1 integrin has also been described as a co-receptor molecule that assists the angiotensin-converting enzyme 2 (ACE2) receptor in the recognition of SARS-CoV-2 infection in Vero E6 cells [13]. Interestingly, Brilha et al. [14] demonstrated that bronchial epithelial cell adherence to a collagen matrix is able to influence matrix metalloproteinase-1 (MMP-1) production and epithelial healing in patients with pulmonary tuberculosis through an α2β1-integrin-dependent manner. On the other hand, a lack of such cell–matrix interactions resulted in the overexpression of MMP-1, which is recognized to be the main factor in tuberculosis immunopathology.

Our group has observed that the α3 and α5 integrins of lung epithelial cells participate in *Paracoccidioides* adhesion and in the induction of IL-6 and IL-8 secretion [15,16]. It was also verified that this fungus promoted an increase in the expression of α3 and α5 integrins in A549 lung epithelial cells during the first 5 h of infection [15]. However, after 24 h infection, *P. brasiliensis* promoted a complete reduction of α3 integrin protein levels in A549 cells, while α5 integrin levels were increased in cells infected with the fungus [16].

In fact, the literature shows that the modulation of integrin levels in cells is very dynamic and complex. There are several steps that control the intracellular trafficking of these receptors, which determine whether they are recycled to the plasma membrane or degraded [17]. Either fate, active or inactive integrins, that are present in the plasma membrane, are primarily endocytosed in clathrin-dependent or independent ways [17,18] and then, they are directed to the early endosomes, which contain small GTPases of the Rab family (Rab5) [17]. The contents of these organelles can be recycled to the plasma membrane, or the early endosomes mature into late endosomes (Rab5 is replaced by Rab7 and Rab25 is recruited), forming the multivesicular bodies that segregate the proteins to the lysosomes for degradation [19,20]. For a successful infection, some pathogens hijack host cellular processes such as vesicular trafficking pathways that promote pathogen entry, replication, or escape [21,22].

As Barros et al. [23] have shown, *P. brasiliensis* promotes the total reduction of α3 integrin protein levels in A549 lung epithelial cells after 24 h of infection. In this work, we investigated the cellular mechanisms involved in the *P. brasiliensis*-induced modulation of α3 integrin levels in epithelial cells.

## 2. Materials and Methods

### 2.1. Epithelial Cells Culture

A549 epithelial cells (human lung adenocarcinoma) were cultured in Dulbecco’s Modified Eagle’s Medium—DMEM (Sigma–Aldrich/Merck, Burlington, MA, USA) supplemented with 10% bovine fetal serum—FBS (Gibco/Thermo Scientific, Waltham, MA, USA), 10 mM HEPES, 100 U/mL penicillin, and 100 μg/mL streptomycin (Sigma-Aldrich/Merck, Burlington, MA, USA) (complete DMEM) at 37 °C, in 5% CO_2_ atmosphere. For *P. brasiliensis*-A549 interaction assays, cells were cultured in 150 mm dishes, 6- or 24-well plates.

### 2.2. Culture and Preparation of P. brasiliensis Yeasts for Interaction Assays with Epithelial Cells

*P. brasiliensis* yeasts, isolate Pb18, were kindly provided by Dr. Wagner Luiz Batista, Universidade Federal de São Paulo (Diadema, SP, Brazil). Fungi were cultured in PGY medium (5 g/L neopeptone, 5 g/L yeast extract, 15 g/L glucose—Becton Dickinson, Franklin Lakes, NJ, USA) containing 1.4 g/L asparagine and 0.1 g/L thiamine (Sigma–Aldrich/Merck, Burlington, MA, USA) for 5–7 days at 37 °C in an incubator at 120 rpm. For the epithelial cell interaction assays, single fungal cells of *P. brasiliensis* were obtained as described by Barros et al. [24]. Briefly, fungi were washed three times with DMEM, kept in this culture medium, and incubated with A549 cells.

### 2.3. Interaction of Epithelial Cells with P. brasiliensis Yeasts

After 48 h of culture, A549 epithelial cells were washed three times with FBS-free DMEM and maintained in this medium at 37 °C and 5% CO_2_. Then, the cells were incubated for different periods with yeasts of *P. brasiliensis* prepared as described in item 2.2. Different multiplicities of infection (MOI = 2.5:1, 1:1, 0.5:1, 0.2:1, 0.1:1) were also performed. In some assays, epithelial cells were pre-incubated with 20 nM bafilomycin A1 (lysosomal acidification inhibitor) or vehicle (0.015% DMSO) (Sigma–Aldrich/Merck, Burlington, MA, USA) for 1 h.

### 2.4. Small Interfering RNA (siRNA)

A549 cells were grown in 6-well plates for 24 h and kept serum-starved for 5 h. Cells were then transfected with a mixture of Lipofectamine RNAiMAX Reagent and Silencer Predesigned siRNA for CHMP3 (Mission^®^ siRNA EHU049141 Sigma/Merk, Burlington, MA, USA) at a final concentration of 10 nM. Negative Control siRNA FLUC (Mission^®^ siRNA EHUFLUC Sigma/Merk, Burlington, MA, USA), which has a sequence that does not target any gene, was used. After 24 h of transfection, cells were washed three times with DMEM and incubated with *P. brasiliensis* yeasts for 24 h (MOI = 1:1). Then, A549 cells were harvested and α3 integrin concentration was analyzed using Western blot. Confirmation of CHMP3 silencing was performed by Reverse Transcription-quantitative Polymerase Chain Reaction (RT-qPCR).

### 2.5. Obtaining Endosome-Enriched Fractions

After the interaction of A549 epithelial cells with *P. brasiliensis* yeasts, protein extracts were obtained by incubating the epithelial cells with the homogenization buffer (250 mM sucrose, 3 mM imidazole pH 7.4, and 1 mM EDTA) containing protease inhibitors (10 µg/mL aprotinin, 500 µg/mL AEBSF, and 10 µg/mL leupeptin) (Sigma–Aldrich/Merck, Burlington, MA, USA). The cell pellet was lysed using a 1 mL syringe and 22 G cannula. Subsequently, an aliquot containing 2 mg of protein was added to the sucrose gradient (42%; 35% and 25%) and ultracentrifuged for 3 h at 4 °C in a Beckman Coulter SW41 Ti rotor 210,000× *g*, as described by the authors of reference [25].

Fractions (1 to 13) of 1 mL each were collected from top to bottom of the tube. Protein content was determined by using the Micro BCA Protein Assay Kit (Pierce/Thermo, Waltham, MA, USA), according to the manufacturer’s instructions and the fractions were analyzed using Western blot. Late endosomes were detected with anti-Rab7 and anti-LAMP-1 antibodies and early endosomes with anti-Rab5 and anti-EEA1 antibodies, as described in item 2.7.

### 2.6. Co-Immunoprecipitation

After incubation of A549 epithelial cells with *P. brasiliensis*, cells were washed with PBS, collected with a cell scraper, and incubated with RIPA lysis buffer (25 mM Tris-HCl pH 7.4 containing 150 mM NaCl, 0.1% sodium dodecyl sulphate—SDS, 1% Triton X-100, and 0.5% sodium deoxycholate) containing protease inhibitors (10 µg/mL aprotinin, 500 µg/mL AEBSF, and 10 µg/mL leupeptin). After 30 min at 4 °C, samples were centrifuged and the protein concentration of the resulting supernatant was quantified using the Micro BCA Protein Assay Kit. Samples containing 500 μg of protein were incubated with 3 μg of anti-α3 integrin antibodies. After 16 h at 4 °C, 20 μL agarose beads conjugated with protein A/G (Santa Cruz Biotechnology, Dallas, TX, USA) were added. After 3 h, agarose beads were washed with RIPA lysis buffer, resuspended in sample buffer (250 mM Tris pH 6.8, 40% glycerol (*w*/*v*), 8% SDS, 0.1% bromophenol blue, and 10% β-mercaptoethanol), boiled for 5 min, and centrifuged. The immunocomplexes were submitted to SDS-PAGE gel (Sodium Dodecyl Sulphate Polyacrylamide Gel Electrophoresis) and analyzed using Western blot with anti-α3 integrin or anti-UbK63 antibodies, as described in item 2.7.

### 2.7. Western Blot

Aliquots containing 20 µg of protein from the cell extracts or 1 μg of protein from endosome-enriched fractions were diluted in sample buffer and submitted to SDS-PAGE. After electrophoresis, proteins were transferred to polyvinylidene difluoride (PVDF) membranes, as described by Maza et al. [24]. Next, PVDF membranes were incubated with: (i) 5% nonfat milk (Cell Signaling Technology, Danvers, MA, USA) in TBST (200 mM Tris-HCl buffer, pH 8.0 with 0.1% Tween-20) for 1 h; (ii) the following primary antibodies diluted in TBST with 1% bovine serum albumin (BSA), overnight at 4 °C—anti-α3 integrin 1:1000 (sc374242, Santa Cruz Biotechnology, Dallas, TX, USA), anti-Rab7 1:1000 (#9367S Cell Signaling Technology, Danvers, MA, USA), anti-Rab5 1:1000 (R4654 Sigma–Aldrich/Merck, Burlington, MA, USA), anti-EEA1 1:1000 (#48453 Cell Signaling Technology, Danvers, MA, USA), anti-LAMP-1 1:1000 (#9091 Cell Signaling Technology, Danvers, MA, USA), anti-UbK63 1:1000 (#5621S Cell Signaling Technology, Danvers, MA, USA), or anti-β-actin 1:15000 (A5441 Sigma–Aldrich/Merck, Burlington, MA, USA); and (iii) secondary antibodies conjugated to horseradish peroxidase (HRP) (anti-mouse IgG #7076 or anti-rabbit IgG #7074 Cell Signaling Technology, Danvers, MA, USA) diluted in 1% BSA in TBST for 1 h at room temperature. After each step, membranes were washed three times with TBST. Finally, PVDF membranes were incubated with Super Signal West Pico Chemiluminescent Substrate^®^ reagent (Pierce/Thermo, Waltham, MA, USA) for 3 min, and recognized proteins by the primary antibodies were detected and documented using the UVITEC photodocumentation system (Cambridge, CAMBS, UK). For quantification of the detected proteins, densitometric analyses of the bands were performed with the Image J V1.8.0.112 software (National Institutes of Health, Bethesda, MD, USA).

### 2.8. Reverse Transcription-Quantitative Polymerase Chain Reaction (RT-qPCR)

After incubation with *P. brasiliensis* yeasts, A549 epithelial cells were washed with PBS to remove the fungi, and total RNA was extracted with RNeasy kit (Qiagen, Hilden, Germany). RNA concentration was determined by measuring absorbance with Nanodrop™ at 260 nm (Thermo/Fisher Scientific, Waltham, MA, USA). For reverse transcription, 2 μg of RNA was incubated with a mixture of the High-Capacity cDNA Reverse Transcription kit, following the manufacturer’s instructions (Applied Biosystems Thermo, Waltham, MA, USA). In parallel, for each sample, a negative control was obtained without the addition of reverse transcriptase enzyme, and later submitted to qPCR in order to evaluate genomic contamination. After obtaining the complementary DNA (cDNA), quantitative PCR was performed by adding 1 μL of the cDNA-containing mixture to a solution containing SYBR Select Master Mix (Applied Biosystems, Waltham, MA, USA). The primers used to analyze the expression of α3 integrin, CHMP3, β-actin, and GAPDH and the size of the expected fragments are described in Table S1. PCR was performed using the Fast cycle on the Step One™ Real Time PCR System (Thermo Fisher Scientific, Waltham, MA, USA). ∆Ct represents the difference of the Ct values of the target gene and housekeeping gene. 2^−∆∆Ct^ was calculated to verify whether the transcription gene of interest was altered by the infection with *P. brasiliensis*. Two targets were tested for housekeeping gene (GAPDH and β-actin), both demonstrated stable and equivalent mRNA expression on A549 cells (data not shown); therefore, GAPDH was used to normalize α3 integrin and β-actin for CHMP3 because of their PCR efficiency similarities among target and housekeeping genes. Target and housekeeping PCR were carried out on the same plate; every plate also included a control (no template) for each primer pair and melting curve analysis for reaction specificity assurance.

### 2.9. Immunofluorescence

After A549 epithelial cells were cultured on glass coverslips (12 mm) for 72 h and infected with yeasts of *P. brasiliensis*, cells were washed three times with PBS, fixed with paraformaldehyde 4% for 20 min, incubated with 50 mM ammonia chloride for 20 min and with PGN/saponin (PBS containing 0.25% gelatin, 0.1% saponin, and 0.1% azide) for 1 h. Subsequently, the coverslips were incubated with the primary antibodies anti-α3 integrin, anti-Rab5 or anti-LAMP-1, diluted 1:50 in PGN/saponin for 2 h at room temperature. The coverslips were then incubated with secondary antibodies conjugated to fluorophores: anti-mouse Alexafluor 488 (#4408S Cell Signaling Technology, Danvers, MA, USA) or Alexafluor 555 anti-rabbit (#4413S Cell Signaling Technology, Danvers, MA, USA). DAPI (4′,6-diamidine-2-phenylindole) (Sigma–Aldrich/Merck, Burlington, MA, USA) was used for nuclear staining and Calcofluor (Sigma–Aldrich/Merck, Burlington, MA, USA) for the staining of the *P. brasiliensis* wall. The coverslips were mounted on glass slides with SlowFade^®^ (S36937, Invitrogen, Waltham, MA USA), and the fluorescence was analyzed with an epifluorescence microscope (Olympus BX51, Tokyo, Japan). Images were taken with the Olympus DP71 camera. Colocalization of α3 integrin and Rab5 or LAMP-1 was analyzed using Image J software (National Institutes of Health, Bethesda, MD, USA).

### 2.10. Cell Viability

The viability of A549 cells was determined using the MTT assay (3-[4,5-dimethylthiazol-2-yl]-2.5 diphenyltetrazolium bromide) as previously described by Maza et al. [26]. Unless otherwise noted in the article, cell viability was examined for each experiment and was found to be greater than 95%.

## 3. Results

### 3.1. Analysis of α3 Integrin Levels in A549 Cells Infected with P. brasiliensis Yeasts

**Figure 1:**
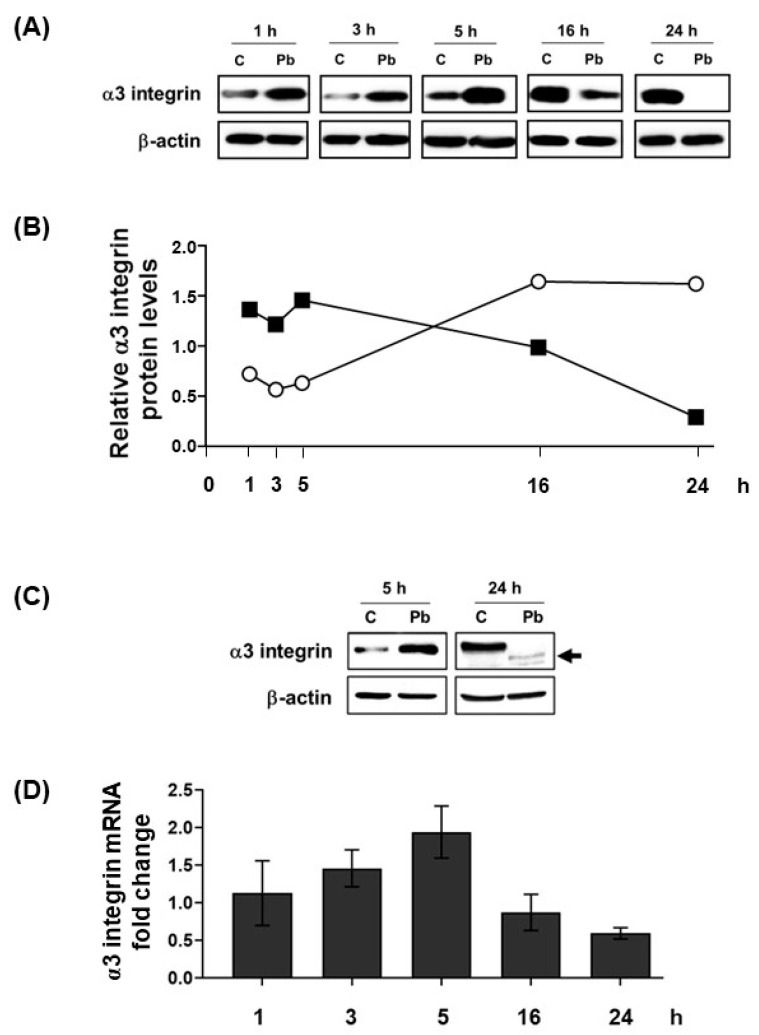
α3 integrin levels in lung epithelial cells during infection with *P. brasiliensis.* Human lung epithelial A549 cells were incubated for different periods with *P. brasiliensis* yeasts (Pb) (MOI 1:1). Control (C) was performed in the absence of yeasts. (**A**) Protein extracts were submitted to SDS-PAGE, and α3 integrin protein levels were analyzed using Western blot. β-actin was used as a loading control. (**B**) Values represent the intensity of the integrin band divided by the corresponding intensity of the β-actin band shown in panel (**A**) (■ Represents infected cells; ○ Represents uninfected cells). (**C**) α3 integrin levels were analyzed using Western blot after 5 h and 24 h infection of A549 cells with *P. brasiliensis*. Arrow points to bands with lower molecular weight than intact α3 integrin (150 kDa). Similar results were obtained in three independent experiments. (**D**) α3 integrin mRNA levels were analyzed via RT-qPCR. GAPDH was used as housekeeping gene. Values represent relative fold change (2^−ΔΔCt^) in target gene transcription levels compared to control sample (without fungi). Mean of three experiments ± standard deviation.

Our group previously described that *P. brasiliensis* yeasts can induce an increase in α3 integrin protein levels in the lung epithelial A549 cell line during the first 5 h of infection. However, surprisingly, after 24 h of incubation with this fungus, the levels of α3 integrin in those epithelial cells were drastically reduced when compared to control cells [15,23]. Corroborating these results, we observed in *P. brasiliensis*-infected A549 cells (Figure 1A,B): (i) at 5 h infection time point, an increase in α3 integrin protein levels in A549 cells up to 2.4-fold when comparing to uninfected cells; and (ii) after 24 h fungal infection, a reduction of this protein levels by 99% in these epithelial cells. The decrease of more than 73% of α3 integrin protein levels in A549 cells was always dependent on the length of infection (24 h) and multiplicity of infection (MOI 1:1) (Figure S1A,B). In these conditions, more than 99% of the A549 cells remained viable throughout the experiment (Figure S1C).

In the current work, while we verified a decrease in α3 integrin levels when comparing *P. brasiliensis*-infected cells within 5 and 16 h time points, uninfected A549 cells presented an increase in α3 integrin levels during this time period (Figure 1A,B). This result indicates that, to reduce α3 integrin levels in A549 cells, *P. brasiliensis* should induce the degradation of this protein and/or promote its transcription decrease. In fact, using Western blot, we observed bands with a lower molecular weight than expected for α3 integrin (150 kDa), which were recognized by anti-α3 integrin antibodies (Figure 1C, arrow), suggesting the existence of degradation products of this protein. α3 integrin transcription levels were also analyzed in A549 cells and we verified that, up to 5 h of infection with *P. brasiliensis*, although not statistically significant, the mRNA levels of this integrin were increased (Figure 1D). However, after 16 h of incubation with *P. brasiliensis*, α3 integrin mRNA was reduced to similar levels to those observed in A549 cells infected for 1 h (*p* = 0.41) (Figure 1D). So, these results indicate that *P. brasiliensis* yeasts can modulate α3 integrin levels in epithelial cells during the course of infection by manipulating different pathways in these cells.

### 3.2. Subcellular Localization of α3 Integrin in A549 Epithelial Cells during Infection with P. brasiliensis

As *P. brasiliensis* yeasts could be inducing α3 integrin degradation in A549 epithelial cells (Figure 1C), we evaluated the involvement of the endolysosomal pathway in this process. In this pathway, proteins are endocytosed, directed to early endosomes and then: (i) the content of these organelles can be recycled back to the plasma membrane; or (ii) the early endosomes mature into late endosomes, which fuse with lysosomes, leading to protein degradation [17]. So, first, we analyzed the subcellular localization of α3 integrin in *P. brasiliensis*-infected A549 cells by indirect immunofluorescence, using antibodies that recognize α3 integrin, the early endosome marker Rab5, or the late endosome/lysosome marker LAMP-1.

**Figure 2:**
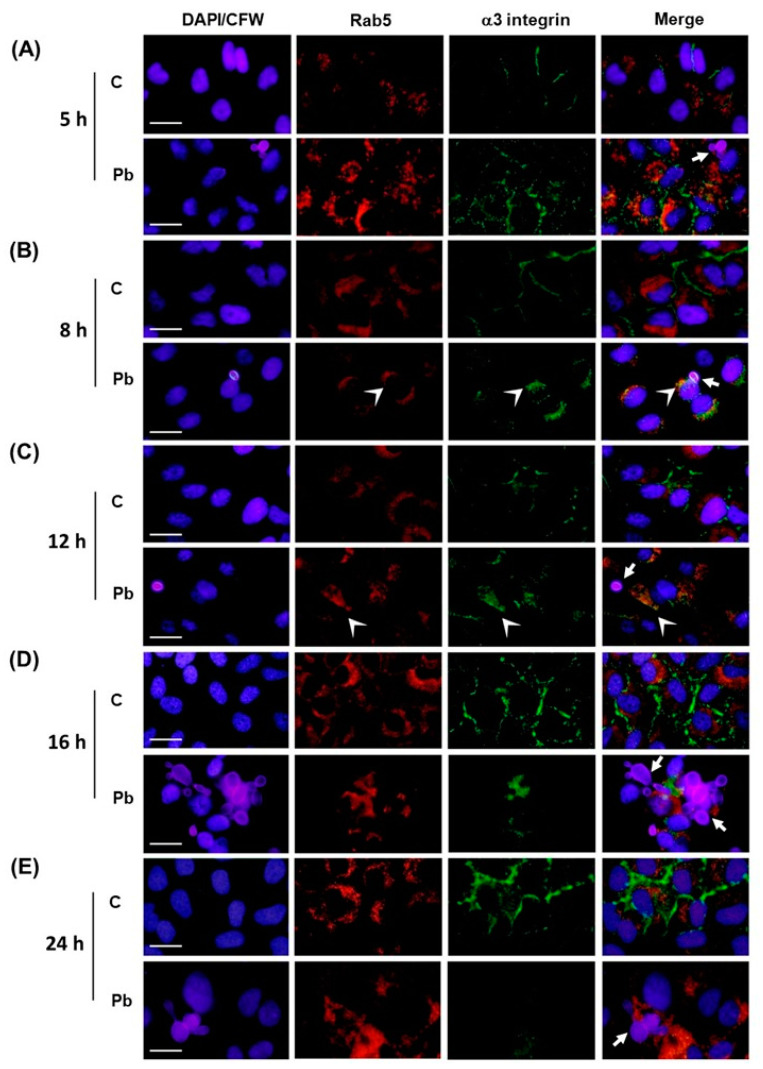
Colocalization analysis of α3 integrin with the early endosome marker Rab5 in A549 epithelial cells infected with *P. brasiliensis.* A549 epithelial cells were infected with *P. brasiliensis* yeasts (MOI 1:1) for (**A**) 5 h, (**B**) 8 h, (**C**) 12 h, (**D**) 16 h, or (**E**) 24 h. Control (C) was performed in the absence of yeasts. Indirect immunofluorescence was performed using anti-α3 integrin antibodies (green) and anti-Rab5 antibodies (red). In blue, the nucleus of A549 cells was stained with DAPI and the fungus cell wall, with calcofluor White. Arrows indicate *P. brasiliensis* yeasts. Colocalization sites of Rab5 and α3 integrin were identified using the ImageJ software and are indicated by the arrowheads. Bar = 20 µm. This result is representative of three independent experiments.

**Figure 3:**
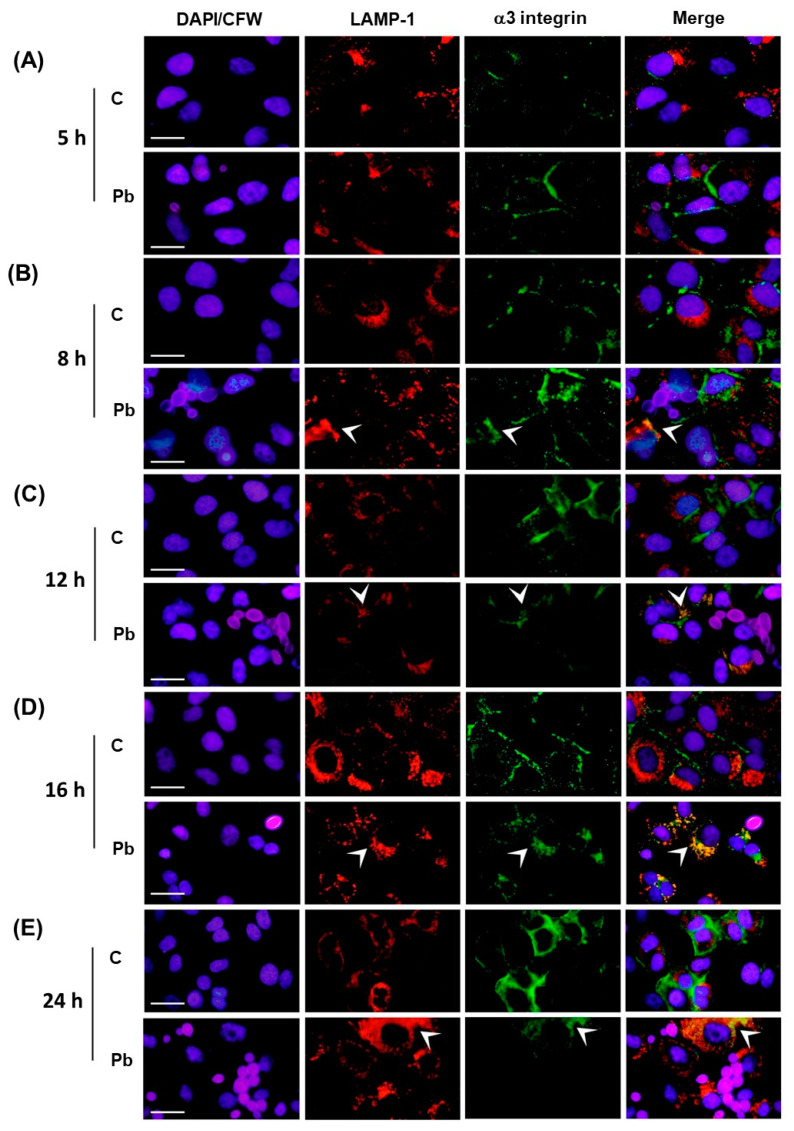
Colocalization analysis of α3 integrin with the late endosome/lysosome marker LAMP-1 in A549 epithelial cells infected with *P. brasiliensis.* A549 epithelial cells were infected with *P. brasiliensis* yeasts (MOI 1:1) for (**A**) 5 h, (**B**) 8 h, (**C**) 12 h, (**D**) 16 h, or (**E**) 24 h. Control (C) was performed in the absence of yeasts. Indirect immunofluorescence was performed using anti-α3 integrin antibodies (green) and anti-LAMP-1 antibodies (red). In blue, the nucleus of A549 cells was stained with DAPI and the fungus cell wall, with calcofluor White. Arrows indicate *P. brasiliensis* yeasts. Colocalization sites of LAMP-1 and α3 integrin were identified using the ImageJ software and are indicated by the arrowheads. Bar = 20 µm. This result is representative of three independent experiments.

Figure 2A,B and Figure 3A,B, show that, in uninfected (control) and *P. brasiliensis*-infected A549 cells at the 5 h point, α3 integrin was found in the cell periphery, while Rab5 and LAMP-1 were found in the A549 cell cytoplasm. No colocalization among these proteins was verified at this time point.

After 8 h of *P. brasiliensis* infection, we verified, in the cytoplasm of some A549 cells, colocalization between Rab5 and α3 integrin (Figure 2B) and also LAMP-1 and α3 integrin (Figure 3B). The colocalization areas of these proteins increased in the A549 cells infected for 12 h with *P. brasiliensis* yeasts (Figure 2 and Figure 3C), indicating the presence of α3 integrin in both early and late endosomes. On the other hand, in control A549 cells, the location of α3 integrin continued in the cell periphery at these time points (Figure 2 and Figure 3B,C).

At the 16 h point of *P. brasiliensis* infection, the colocalization areas of α3 integrin and Rab5 decreased in infected A549 cells (Figure 2D). On the other hand, we verified the highest amount of colocalization between α3 integrin and LAMP-1, indicating the presence of α3 integrin in late endosomes/lysosomes at the 16 h point of infection (Figure 3D).

Corroborating the Western blot results (Figure 1), a weak or no immunofluorescence signal with anti-α3 integrin antibodies was verified in A549 cells infected for 24 h with *P. brasiliensis* (Figure 2 and Figure 3). At this time point, uninfected (control) A549 cells still presented high levels of α3 integrin immunofluorescence in the periphery of A549 cells (Figure 2 and Figure 3E).

### 3.3. Presence of α3 Integrin in Late Endosome-Enriched Fractions of Epithelial Cells during P. brasiliensis Infection

Next, to corroborate the presence of α3 integrin in the epithelial cell late endosomes/lysosomes, *P. brasiliensis*-infected A549 cell lysates were submitted to a subcellular fractionation protocol, using sucrose gradient and ultracentrifugation [25]. For this assay, a period of 16 h of fungal-epithelial cell incubation was chosen because it was the longest period of infection with detectable levels of α3 integrin protein and with the highest colocalization between α3 integrin and LAMP-1 (Figure 3D).

After A549 cell fungal infection, cell lysis, and ultracentrifugation, 13 fractions of 1 mL each were collected, submitted to Western blot, and the presence or not of α3 integrin, the early endosome marker EEA1, and the late endosome/lysosome marker LAMP-1 was analyzed. Figure S2 shows a typical subcellular fractionation of A549 cells obtained by our group. We verified that fractions 1 and 2 were enriched in late endosomes, since LAMP-1 was present and EEA1 was absent. However, we were unable to obtain fractions enriched in early endosomes, because fractions 3 to 7 presented not only EEA1, but also LAMP-1, indicating the presence of both early and late endosomes. Figure S2 also shows that α3 integrin was present in fractions 1 to 6.

**Figure 4:**
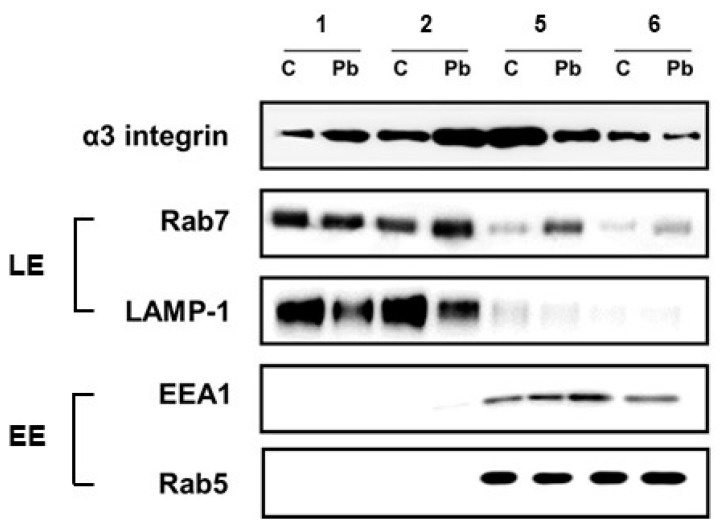
α3 integrin levels in endosome-enriched fractions of A549 epithelial cells infected with *P. brasiliensis.* A549 cells were incubated in the absence (C) or presence of *P. brasiliensis* yeasts (Pb) for 16 h (MOI 1:1). Then, the epithelial cells were collected, lysed, and submitted to sucrose density gradient/ultracentrifugation. Aliquots of endosome-enriched fractions 1, 2, 5, and 6 were submitted side by side to the same SDS-PAGE. Next, Western blot was performed using antibodies anti-α3 integrin, -EEA1, -Rab5, -Rab7, and -LAMP-1. LE: Late endosome markers; EE: Early endosome markers. This result is representative of three independent experiments.

To compare α3 integrin levels in endosome-containing fractions of *P. brasiliensis*-infected and uninfected (control) A549 cells, aliquots of these fractions were submitted side by side to the same SDS-PAGE/Western blot. Corroborating Figure S2 results, Figure 4 shows that fractions 1 and 2 are enriched in late endosomes/lysosomes due to the presence of the late endosome markers Rab7 and LAMP-1 and the absence of the early endosome markers EEA1 and Rab5. On the other hand, even though fractions 5 and 6 were enriched in early endosomes (presence of EEA1 and Rab5 markers), we also verified that these fractions contain the late endosome markers Rab7 and LAMP-1, indicating the presence of both kinds of endosomes in these samples. Figure 4 also shows that, after 16 h of infection, *P. brasiliensis* yeasts promoted in A549 cells an increase in α3 integrin levels in fractions 1 and 2 (up to 2.2-fold) when compared to uninfected cells, indicating that the fungus induced α3 integrin sorting to late endosomes and lysosomes in A549 epithelial cells at this time point.

### 3.4. α3 Integrin Levels in CHMP3-Silenced A549 Epithelial Cells Infected with P. brasiliensis

**Figure 5:**
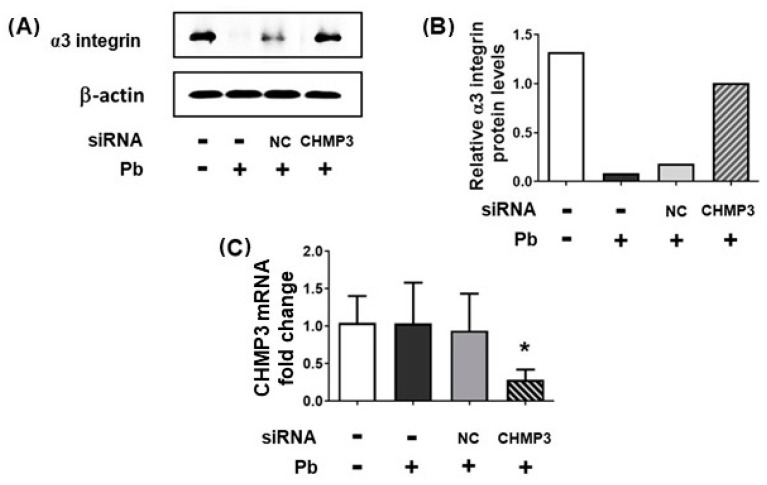
Effect of CHMP3 silencing on the α3 integrin levels during infection of A549 epithelial cells with *P. brasiliensis*. A549 cells were transfected with siRNA directed to CHMP3 (CHMP3) or negative control (NC) siRNA. Next, A549 cells were infected (+) or not (−) with *P. brasiliensis* yeasts (Pb) (MOI 1:1) for 24 h. (**A**) Protein extract aliquots were submitted to SDS-PAGE and α3 integrin levels were analyzed using Western blot. β-actin was used as loading protein control. (**B**) Values represent the intensity of the integrin band divided by the intensity of the corresponding β-actin band shown in (**A**). (**C**) CHMP3 silencing was analyzed by RT-qPCR. β-actin was used as housekeeping gene. Values represent relative fold change (2^−ΔΔCt^) in the target gene transcription levels compared to control group (without siRNA and Pb). Mean of triplicates ± standard deviation. *, *p* < 0.01 when compared to NC. Similar results were obtained in two independent experiments.

An important step for the maturation of early to late endosomes is the increase in intraluminal vesicles [27]. This process is carried out by the proteins of the endosomal sorting complex required for transport (ESCRT) and the charged multivesicular body protein 3 (CHMP3) is one of the proteins that forms the ESCRT-III complex involved in intraluminal vesicle membrane remodeling and scission [28,29]. So, CHMP3 was silenced in A549 cells and α3 integrin levels were analyzed after 24 h infection with *P. brasiliensis*. Under these conditions, we observed that the fungus was able to reduce only 23.4% of α3 integrin levels when compared to uninfected cells (Figure 5A,B). On the other hand, *P. brasiliensis* promoted an 84.8% integrin level decrease in negative control (NC) siRNA-transfected A549 cells when compared to uninfected cells (Figure 5A,B). Therefore, these results indicate that *P. brasiliensis* yeasts can induce the sorting of α3 integrin to the endolysosomal pathway in A549 epithelial cells.

CHMP3 silencing was confirmed using RT-qPCR (Figure 5C). We observed that, after 24 h, CHMP3 mRNA levels were reduced by 70.2% in CHMP3-silenced A549 cells when compared to NC siRNA-transfected A549 cells (Figure 5C).

### 3.5. Analysis of α3 Integrin Levels in A549 Epithelial Cells during Infection with P. brasiliensis in the Presence of Bafilomycin A1

**Figure 6:**
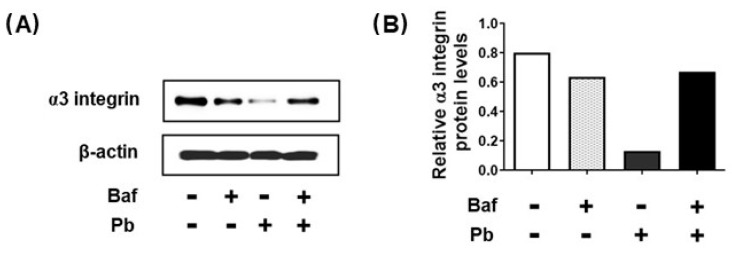
α3 integrin levels during infection with *P. brasiliensis* in the presence of bafilomycin A1. A549 cells were pre-incubated for 1 h with 20 nM of bafilomycin A1 (Baf+) and subsequently with *P. brasiliensis* yeasts (Pb) (MOI 1:1) for 24 h. Control (C) was performed in the absence of yeasts (−). (**A**) Protein extracts were submitted to SDS-PAGE and α3 integrin levels were analyzed using Western blot. β-actin was used for sample normalization. (**B**) Values represent the intensity of the α3 integrin band divided by the intensity of the corresponding β-actin band. Similar results were obtained in three independent experiments.

Bafilomycin A1 is an inhibitor of vacuolar H+-ATPase and, consequently, it inhibits endosome maturation and fusion with lysosomes [30]. In this manner, we incubated A549 cells with 20 nM bafilomycin A1 and then infected with *P. brasiliensis* for 24 h. Figure 6 shows that *P. brasiliensis* (Pb) yeasts were not able to reduce the α3 integrin levels in bafilomycin-treated A549 cells. In fact, α3 integrin levels were 5.1-fold higher under these conditions (Baf+ Pb+), when compared to untreated epithelial cells infected with the fungus (Baf− Pb+) (Figure 6A,B). Therefore, these results indicate that *P. brasiliensis* yeasts promote the degradation of α3 integrin through the endolysosomal pathway in epithelial cells.

## 4. Discussion

Integrins are host cell receptors that have been linked to a variety of cellular functions, including cytoskeletal organization, cell proliferation, adhesion, differentiation, and migration [31]. Furthermore, during an infectious event, these receptors play a crucial role in the adherence and/or invasion of several viruses, bacteria, and fungi into the host cell, and they may also contribute to the host inflammatory response [13,15,16,32,33,34,35].

The goal of our research group has been to understand the responses of human lung epithelial cells after interaction with the human respiratory pathogenic fungi *Paracoccidioides* and *Histoplasma capsulatum* [4,15,16,23,24,26,36,37,38,39]. We have observed over the years that the integrin family receptors of lung epithelial cells participate in fungal adhesion and cytokine secretion during interaction with *Paracoccidioides* yeasts [15,16,23]. Intriguingly, we also verified that *P. brasiliensis* completely decreased the levels of α3 integrin in A549 epithelial cells following a 24 h infection [16]. In fact, in this study, the results from the incubation of A549 epithelial cells with *P. brasiliensis* and the lysosomal acidification inhibitor bafilomycin A1 clearly demonstrated that the degradation of α3 integrin occurs via lysosomes because there was no decrease in the protein levels of α3 integrin under these conditions.

In fact, an usual pathway for integrins after endocytosis is recycling to the plasma membrane, where they can interact with new ligands [40]. On the other hand, non-recycled integrins are sorted into the endolysosomal pathway [41,42], and, according to the results of this work, *P. brasiliensis* yeasts promoted α3 integrin degradation through this mechanism in A549 epithelial cells. The indirect immunofluorescence results, for example, showed an increase in colocalization between α3 integrin and the marker LAMP-1 after 16 h of *P. brasiliensis* infection, indicating the presence of α3 integrin in late endosomes/lysosomes. By using a subcellular fractionation approach, we were also able to confirm this by verifying an increase in α3 integrin levels in the late endosome fractions obtained from A549 epithelial cells after infection with *P. brasiliensis*.

Additionally, in CHMP3-silenced A549 epithelial cells, *P. brasiliensis* was able to promote only a small decrease in α3 integrin levels. This finding was important because CHMP3 is a protein that is crucial for the functioning of the ESCRT-III machinery, ILV formation, and maturation of early to late endosomes [43,44]. Some groups have also demonstrated that the silencing of CHMP3 (ESCRT-III) inhibits the receptor degradation through the lysosomal pathway [45,46]. Furthermore, lysine 63 (K63)-linked protein ubiquitination may also be associated with the sorting of these molecules for degradation via the endolysosomal pathway [47,48,49]. In fact, we found that *P. brasiliensis* infection for 16 h increased the levels of K63-linked ubiquitination of the α3 integrin in A549 epithelial cells, corroborating the fact that, at intermediate periods (from 8 to 16 h), α3 integrin is endocytosed and forwarded to early and late endosomes (Figure S3).

Thus, taken together, the results of this study clearly demonstrate that *P. brasiliensis* yeasts can stimulate α3 integrin reduction levels in lung epithelial cells primarily by hijacking host cell trafficking, as we observed a major involvement of the endolysosomal pathway for the degradation of this receptor rather than a complete inhibition of α3 integrin transcription.

Studies on the negative modulation of integrins during the migration, progression, and metastasis of tumor cells have been published by some groups [50,51,52], and few studies have shown how pathogens might induce the decrease in integrin levels in host cells by either reducing the transcription levels or inducing protein degradation. He et al. [53], for example, showed that the hepatitis B virus X protein (HBx) promoted a decrease in the transcription and protein levels of the α3 integrin in murine podocyte cell line, altering cell adhesion. Additionally, in an elegant study by Thuenaver et al. [54], it was demonstrated that the *Pseudomonas aeruginosa* lectin LecB binds to integrins only when basolateral cell surface is available, leading to integrin internalization and degradation, which facilitates this bacterial infection.

Even though we observed that *P. brasiliensis* promoted the negative modulation of α3 integrin in epithelial cells, the response of epithelial cells to an infection is complex. It is also noteworthy to highlight that our previous work has demonstrated that *P. brasiliensis* increased α3 integrin levels in A549 cells at the initial stage of the infection (5 h) and promoted interaction between this receptor and Toll-like receptor 2 (TLR2) [23]. However, as was also demonstrated in the current work, we unexpectedly found that, after 24 h, there was a reduction in α3 integrin levels that was dependent on TLR2 and direct contact between fungi and epithelial cells [23]. So, more research is required to comprehend the consequences of these host cellular events during epithelial cell infection by *Paracoccidioides*.

Despite this, α3β1 integrin has been described as a crucial molecule for the establishment and maintenance of epithelial tissues [55,56]. In fact, some studies have reported that the α3β1 integrin gene ITGA3 loss-of-function or mutations lead to a rare multi-organ disorder, and some patients may present severe dysfunction and inflammation in the lungs and kidneys [57,58]. In experimental models, a study carried out by Kim et al. [59] demonstrated that α3β1 integrin plays a role in murine pulmonary fibrosis. The authors showed that when murine epithelial cells were silenced for α3 integrin and stimulated with bleomycin (antineoplastic drug with fibrotic effects), the lung tissue of mice showed a reduced accumulation of myofibroblasts and type I collagen and did not progress to pulmonary fibrosis. Considering that the absence of pulmonary fibrosis is observed in acute cases of PCM, a more severe form of this mycosis [60], it is possible that the reduction in α3 integrin in pulmonary epithelial cells promoted by *P. brasiliensis* is involved in the pathogenesis of the acute form. However, further studies are needed to elucidate the impact of the negative regulation of α3 integrin on epithelial cells during *P. brasiliensis* infection.

## Data Availability

Not applicable.

## Supplementary Materials

The following supporting information can be downloaded at: https://www.mdpi.com/article/10.3390/jof9090912/s1, Figure S1: α3 integrin levels in A549 cells infected with different MOIs of *P. brasiliensis*; Figure S2: Analysis of early and late endosomes in the fractions obtained by sucrose density gradient/ultracentrifugation; Figure S3: Analysis of K63-linked ubiquitinated α3 integrin in A549 epithelial cells infected with *P. brasiliensis* yeasts; Table S1: Primer sequences used in real time quantitative PCR analysis.
