# Supplementary material for: Paracoccidioides brasiliensis Induces α3 Integrin Lysosomal Degradation in Lung Epithelial Cells
Source: J Fungi (Basel). 2023 Sep 8;9(9):912. doi: 10.3390/jof9090912 (PMC10532483; doi:10.3390/jof9090912)
Supplement: Supplementary file 1 [file jof-09-00912-s001.zip › jof-2585007-supplementary.pdf]

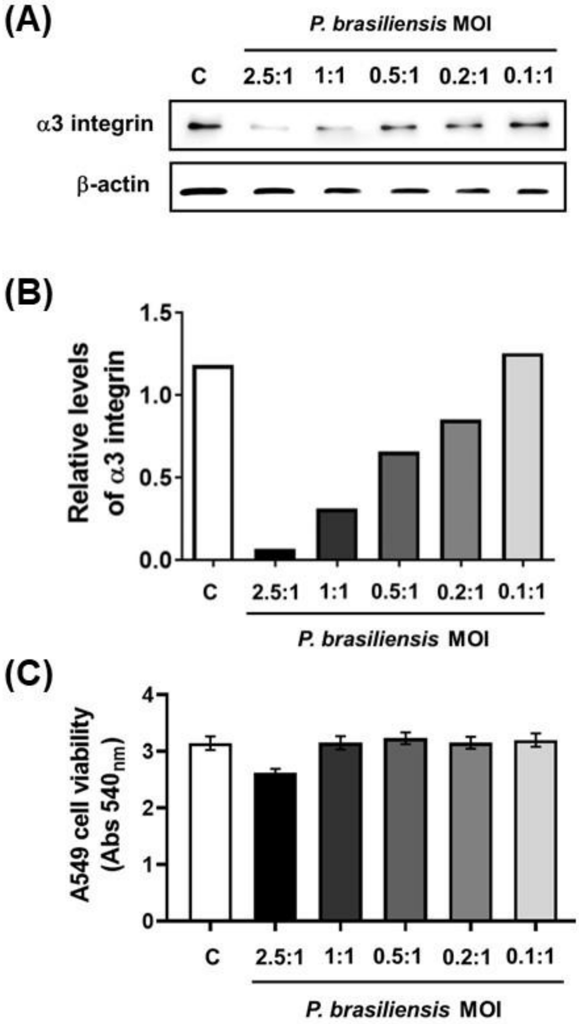

Figure S1. α3 integrin levels in A549 cells infected with different MOIs of *P. brasiliensis*

Different ratios of *P. brasiliensis* yeasts were incubated with A549 epithelial cells for 24 h (MOIs 2.5:1; 1:1; 0.5:1; 0.25:1, and 0.1:1). Control (C) was performed in the absence of yeasts. **(A)** Protein extracts were submitted to SDS-PAGE and *α*3 integrin levels were analyzed by Western blot. β-actin was used as loading control. **(B)** Values represent the intensity of the integrin band divided by the intensity of the corresponding β-actin. **(C)** Viability of A549 cells infected with *P. brasiliensis* yeasts was determined by MTT cell viability assay. Similar results were obtained in three independent experiments.

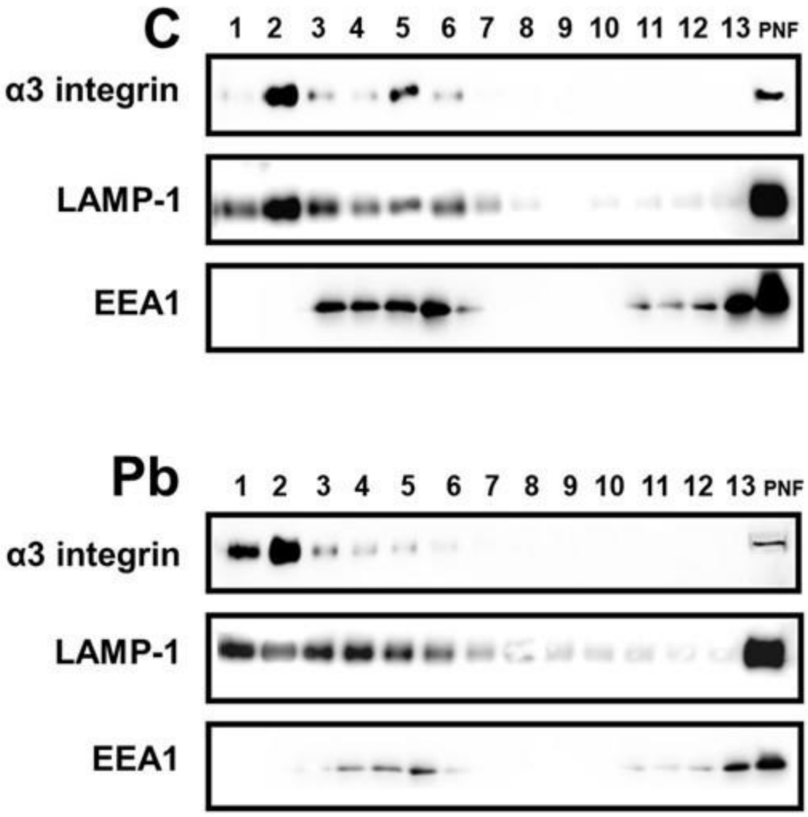

Figure S2. Analysis of early and late endosomes in the fractions obtained by sucrose density gradient/ultracentrifugation

A549 cells were incubated in the absence (C) or presence of *P. brasiliensis* yeasts (Pb) for 16 h (MOI 1:1). Then, the cells were collected and lysed. Same amount of protein of the post-nuclear fraction (PNF) was submitted to the sucrose density gradient and, after ultracentrifugation, 13 fractions of 1 mL were collected. Aliquots containing 1 μg of protein from these fractions and 20 μg of PNF were submitted to SDS-PAGE and integrin α3, LAMP-1 (a marker of late endosome/lysosome), and EEA1 (a marker of early endosome) were analyzed by Western blot. Similar results were obtained in three independent experiments.

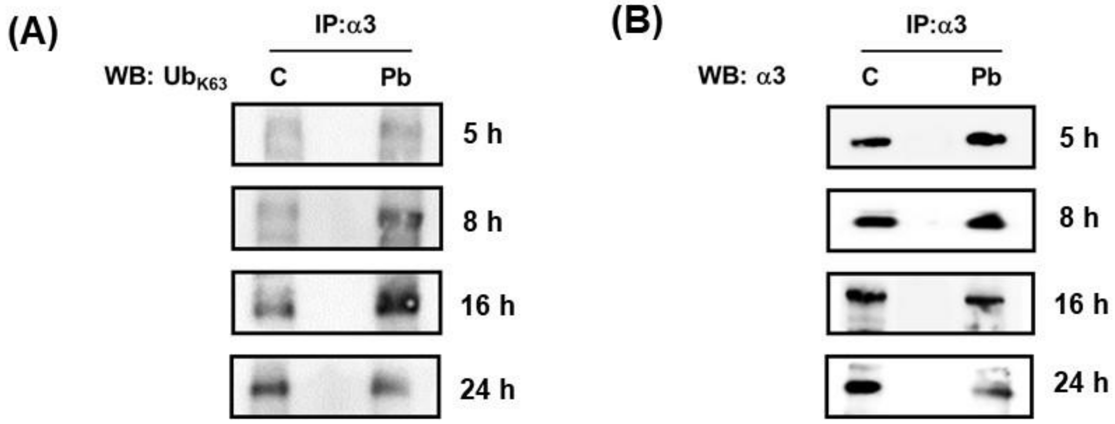

Figure S3. Analysis of K63-linked ubiquitinated *α*3 integrin in A549 epithelial cells infected with *P. brasiliensis* yeasts

A549 cells were infected with *P. brasiliensis* yeasts (MOI 1:1). After 5, 8, 16, or 24 h, epithelial cells were lysed and aliquots containing 300 µg of protein were incubated with anti-*α*3 integrin antibodies (IP). Then, agarose beads conjugated to A/G protein were added. Immunoprecipitates were analyzed by Western blot (WB), using antibodies (**A)** anti- UbK63 or **(B)** anti-*α*3 **i**ntegrin. This result is representative of two independent experiments.

Table S1. Primer sequences used in real time quantitative PCR analysis

Gene	Sequence (5′→ 3′)	Concetration (μM)	Expected PCR product size
α3 integrin	Forward: TGCCTACAACTGGAAAGGAAAC<br>Reverse: CTGCCTACCTGCATCGTGTA	0.25<br>0.25	126 bp
CHMP3	Forward: TGATCAGGTCAAGGAAGGCCA<br>Reverse: CACTTTACTGGGTGCTTTGCC	0.25<br>0.25	166 bp
β-actin	Forward: CACCATTGGCAATGAGCGGTTC<br>Reverse: AGGTCTTTGCGGATGTCCACGT	0.5<br>0.5	135 bp
GAPDH	Forward: GTCTCCTCTGACTTCAACAGCG<br>Reverse: ACCACCCTGTTGCTGTAGCCAA	0.25<br>0.12	131 bp
